# Physiotherapy Approach for Treating Bronchopneumonia: A Case Report

**DOI:** 10.7759/cureus.51246

**Published:** 2023-12-28

**Authors:** Aakanksha Zade, Aditi Akhuj, Lajwanti Lalwani, Saurabh Jhunjhunwala, Ritik V Daf

**Affiliations:** 1 Cardiovascular and Respiratory Physiotherapy, Ravi Nair Physiotherapy College, Datta Meghe Institute of Higher Education and Research, Wardha, IND

**Keywords:** airway clearance technique, active breathing exercises, modified medical research council, pulmonary rehabilitation, bronchopneumonia

## Abstract

The term bronchopneumonia describes an inflammation of the bronchioles centered in the lungs. A male patient, aged 77, complained of dyspnea for six months. The Modified Medical Research Council (mMRC) Dyspnea Scale showed grade 2 dyspnea, chest pain, cold, and fever for seven days. X-rays were done that revealed bronchopneumonia. The research aimed to understand the effect of chest physical therapy in patients admitted to high-density units. We, as physiotherapists, use a wide range of treatments, such as airway clearance procedures, early mobility, and active breathing exercises, all of which are useful in reducing the symptoms of pneumonia in this situation. The outcome measures used were the mMRC Dyspnea Scale, Intensive Care Unit (ICU) Mobility Scale, Functional Independence Measure (FIM), and Numerical Pain Rating Scale (NPRS). Early physiotherapy rehabilitation is beneficial in resolving bronchopneumonia and relieving dyspnea.

## Introduction

Pneumonia is a restrictive lung disease that develops when the lungs become infected by bacteria, fungi, or viruses. A form of pneumonia called bronchopneumonia causes inflammation in the lungs. The term "bronchopneumonia" describes an inflammation of the lung centered in the bronchioles and tiny air sacs. Based on data from the 2019 Global Burden of Diseases Research, 489 million people worldwide suffer from lower pneumonia and bronchitis, which are often known as lower respiratory tract infections. Among the primary reasons for infection-related mortality and morbidity in older people is pneumonia [1]. It is a potentially fatal illness caused by inhaled bacteria and viruses, lower respiratory tract inflammation, and infection of alveoli and bronchioles. Bacteria can cause two types of pneumonia, gram-negative and gram-positive. Gram-positive pneumonia can be brought on by various kinds of bacteria, including *Enterococcus*,* Staphylococcus aureus*,* Streptococcus pneumoniae *(*pneumococcus*), and *Streptococcus pyogenes*. *Pseudomonas aeruginosa*,* Klebsiella pneumoniae*, and *Haemophilus influenzae* cause gram-negative pneumonia. Fungal-induced pneumonia is less common, but it can occur in patients taking immunosuppressive drugs, suffering from acquired immune deficiency syndrome, or having other immune system disorders [2,3]. The disease typically presents as painful chest discomfort and a chronic cough that discharges copious amounts of mucous. This inflammation results in the generation of mucopurulent exudates, which block some tiny airways and cause the neighbouring lobules to consolidate unevenly. In addition to the chest X-ray showing the presence of a fresh infiltration, respiratory clinical signs like fever, cough, and dyspnea are employed in the finding of a pneumonia case. Diagnosing this is more challenging for elderly individuals because of age-related abnormal symptoms, such as a decrease in the threshold for fever. Patients frequently arrive in the emergency room due to falls or concurrent comorbidity decompensation [4]. When diagnosing pneumonia in those over 65, a low-dose computed tomography scan is a more suitable method than a high-quality chest X-ray due to its ease of use and interpretation [5]. Every year, bronchopneumonia claims the lives of up to 5 billion children under the age of five in developing nations. The main cause of this acute viral illness is the respiratory syncytial virus. Illness is characterized by hyperinflation, wheezing, and tiny inspiratory crackles [6]. General hygiene practices, such as mask-wearing, contact, and droplet prevention, are essential instruments to fight against respiratory illnesses worldwide, including viral infections [7].

Prolonged rest by elderly adults suffering from pneumonia often results in long-term recumbency and a reduction in day-to-day activity; early rehabilitation benefits the respiratory, cardiovascular, and locomotor systems as well as the mental condition of patients in bed. Promoting and carrying out rehabilitation in these people requires adherence to clinical and rehabilitation criteria. The primary goal of physical therapy (PT) is to maintain the airways' normal opening and function [8]. While there are criteria for rehabilitation specific to different lung diseases, it is unclear if the same recommendations and results would hold for those with bronchopneumonia [9].

## Case presentation

Patient information

A 77-year-old male patient visited Acharya Vinoba Bhave Rural Hospital with complaints of breathlessness for six months. The Modified Medical Research Council (mMRC) Dyspnea Scale showed grade 2 dyspnea (walks slower than people of the same age because of dyspnea or has to stop for breath when walking at own pace), chest pain, cold, and fever seven days back. Investigations like complete blood tests, sputum cultures, and X-rays were done. Blood investigation revealed reduced white blood cell (WBC) and platelet count, and sputum culture showed no pathogenic organism, whereas Ziehl-Neelsen (ZN) staining was negative for acid-fast bacilli, and an X-ray suggested bronchopneumonia. After being hospitalized in the intensive care unit (ICU), the patient received bilevel-positive airway pressure (BiPAP) support at a fraction of inspired oxygen (FIO2) of 40%. He was a chronic smoker and alcoholic for 50 years. The patient was on medication Doxovent 400 mg (Glenmark Pharmaceuticals, Mumbai, India), Mucinac 600 mg (Cipla, Mumbai, India), and injection Hydrocort 100 mg (Abbott Laboratories, Chicago, Illinois, United States). No other history of illness and surgery was found. He was referred for physiotherapy on October 10, 2023, and a carefully planned physiotherapeutic protocol was initiated.

Clinical findings

The patient's informed permission was obtained before the examination. The patient was examined in a supine lying position. He was conscious and well-oriented to time, place, and person and was hemodynamically stable. Arterial blood gas (ABG) analysis findings are depicted in Table 1. Table 2 shows the FIO2 and positive end-expiratory pressure (PEEP). On observation, chest symmetry was seen as bilaterally symmetrical, but reduced chest movements and use of accessory muscles were noted during breathing. On auscultation, bilateral crepitus was heard in the lower zones, and air entry was also reduced. Table 3 shows the patient's timeline.

**Table 1:** Pre-intervention ABG analysis ABG: arterial blood gas; pH: potential of hydrogen; PCO2: partial pressure of carbon dioxide; PO2: partial pressure of oxygen; HCO3: bicarbonate Reference values: pH: 7.34-7.45; PCO2: 35-45 mmHg; PO2: 75-100 mmHg; HCO3: 22-26 mmol/L

Sr. no.	Dates	ABG findings	Interpretation
1	15/10/23	pH: 7.22; PCO2: 38 mmHg; PO2: 72 mmHg; HCO3: 22 mmol/L	Metabolic acidosis
2	16/10/23	pH: 7.32; PCO2: 37 mmHg; PO2: 70.8 mmHg; HCO3: 24 mmol/L	Metabolic acidosis
3	17/10/23	pH: 7.34; PCO2: 43 mmHg; PO2: 99 mmHg; HCO3: 26 mmol/L	Normal
4	18/10/23	pH: 7.43; PCO2: 44 mmHg; PO2: 100 mmHg; HCO3: 22.7 mmol/L	Normal
5	19/10/23	pH: 7.42; PCO2: 43 mmHg; PO2: 100 mmHg; HCO3: 24.4 mmol/L	Normal

**Table 2:** Pre-intervention FIO2 and PEEP FIO2: fraction of inspired oxygen; PEEP: positive end-expiratory pressure

Sr. no.	Date	FIO2%	PEEP
1	15/10/23	40%	5 cmH2O
2	16/10/23	40%	5 cmH2O
3	17/10/23	40%	5 cmH2O
4	18/10/23	30%	5 cmH2O
5	19/10/23	30%	5 cmH2O

**Table 3:** Timeline of the patient from the date of admission to follow-up

Events	Date
Date of admission	08/10/23
Date of commencement of physiotherapy rehabilitation	12/10/23
Date of discharge	23/10/23

Diagnostic assessment 

**Figure 1:**
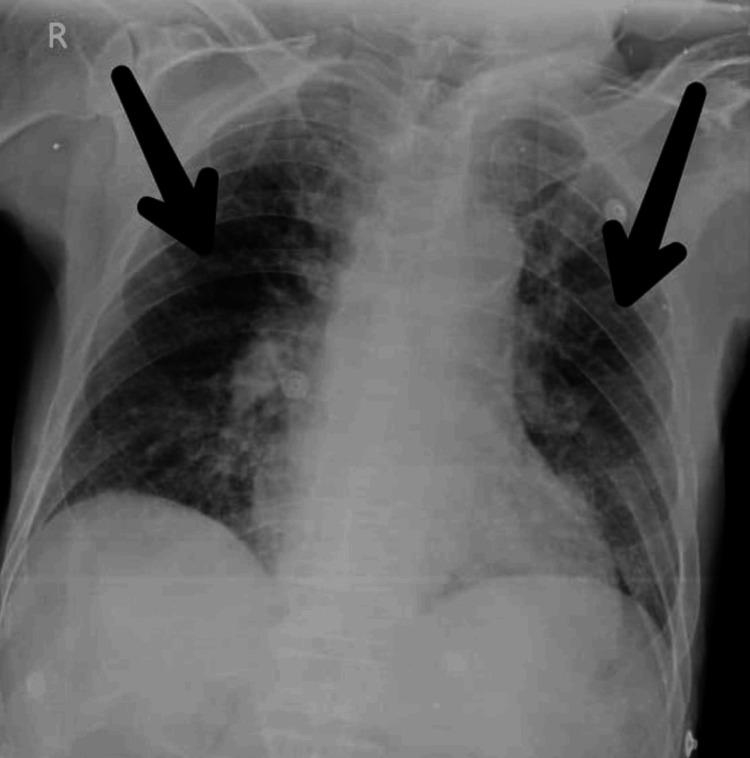
Pre-intervention chest X-ray in PA view PA: posteroanterior The black arrow points at prominent bronchovesicular markings

**Figure 2:**
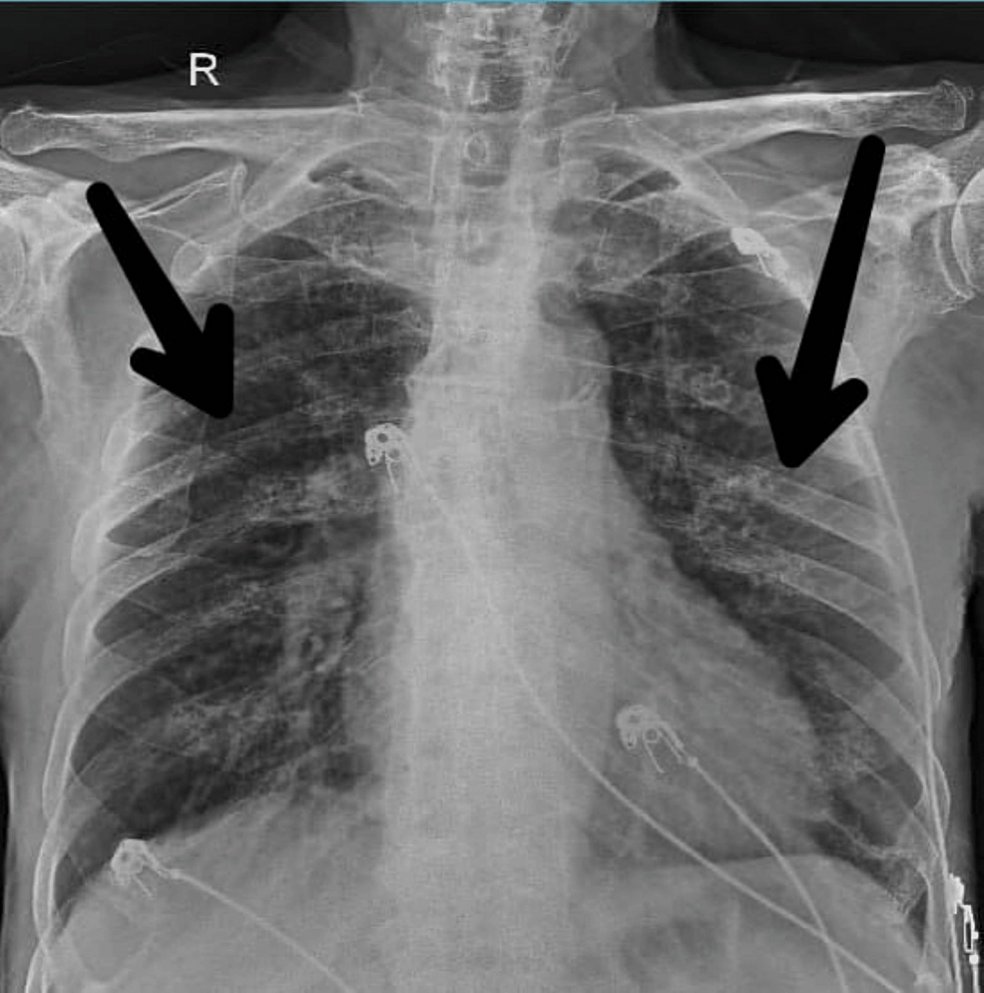
Chest X-ray in PA view showing relatively clear lungs post intervention PA: posteroanterior

A chest X-ray was done, which revealed a hypertranslucent lung bilaterally with visible bronchovesicular markings pre intervention (Figure 1). Figure 2 shows the post-intervention (after four weeks) X-ray scan which reveals clear lung zones bilaterally.

Therapeutic intervention 

**Figure 3:**
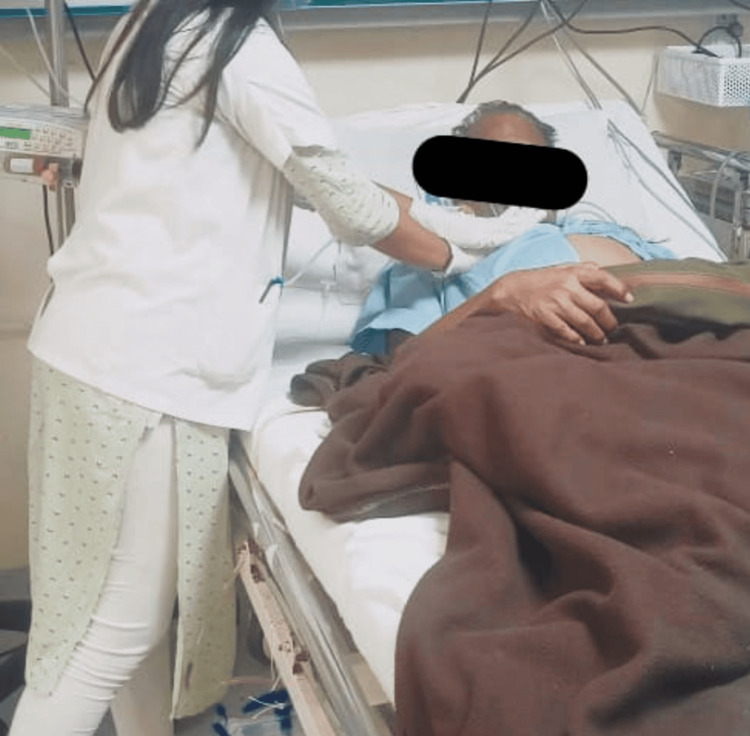
Therapist performing manual chest vibration

The mainstay of treatment for pneumonia is antibiotics; other medicines provide supportive care. Additional oxygen, intravenous hydration, and chest PT are examples of these adjuvant therapies. Chest physiotherapy is a method of clearing the airways that includes teaching cough and breathing methods and strategically placing the patient to allow for mucus drainage. Vibrations, coughing, postural drainage, and percussion are all part of chest physiotherapy done to remove the secretions from the respiratory tract. Active breathing control, forced expiration method, thoracic expansion exercises, and bilateral upper limb and lower limb mobility exercises are done to improve the range of motion of various joints. Table 4 and Figure 3 show the therapeutic intervention given to the patient.

**Table 4:** Physiotherapy intervention ACBT: active cycle of breathing techniques; B/L: bilateral; UL: upper limb; LL: lower limb, reps: repetition

Sr. no.	Goals	Physiotherapy intervention	Rationale
1	Patient-family education and counselling	To inform the patient about their current condition as well as the advantages of a physiotherapy intervention and regimen	Aids in improving the patient's understanding of the disease in order to motivate active engagement and boost treatment effectiveness
2	To remove secretions and to keep up good bronchial hygiene	Vibration, coughing, suctioning, and nebulization with Budecort (Cipla, Mumbai, India)	Assists in clearing airway blockage, tracheobronchial secretions, and inflammatory exudates and also lessens airway resistance, which facilitates better breathing and gas exchange
3	To improve respiratory function	Deep breathing (10 reps, 2 sets), thoracic expansion (10 reps, 1 set), ACBT (3 cycles)	Enhances airflow to all lung segments and dislodges secretions
4	To improve the range of motion of joints	B/L UL and LL exercises	Helps strengthen the lungs and restore function
5	To improve balance and feeling of independence	Ambulation	Helps in joint flexibility and muscle mass and strength
6	To improve the quality of life of a patient	Deep breathing techniques, upper and lower limb mobility exercises, and ambulation are all included in the home exercise program	Sustains the improvement and encourages more advancement

Follow-up and outcome measures

Mortality and length of stay were the main outcome measures. Secondary end measures included physical function, exercise capacity, symptoms, and quality of life assessments, along with the risk of hospital readmissions and emergency visits. After four weeks of treatment protocol, on auscultation, air entry was bilaterally equal, and no adventitious sounds were heard. The patient was on room air. Table 5 shows the outcome measures of the patient.

**Table 5:** Outcome measures NPRS: Numerical Pain Rating Scale; mMRC: Modified Medical Research Council; ICU: Intensive Care Unit mMRC Dyspnea Scale: Grade 1: short of breath when hurrying or walking up the slight hill; Grade 2: walk slower than contemporaries on the level because of breathlessness or has to stop for breath when walking at one pace ICU Mobility Scale: Grade 1: sitting in bed and exercise in bed; Grade 8: walking with the assistance of one person

Outcome measures	Pre treatment	Post treatment
NPRS	On rest: 4/10; on activity: 7/10	On rest: 1/10; on activity: 3/10
mMRC Dyspnea Scale	Grade 2	Grade 1
ICU Mobility Scale	Grade 1	Grade 9
Functional Independence Measure	22/126	100/126

## Discussion

Across all age categories, pneumonia is one of the most prevalent health problems in the globe. The core of therapy for pneumonia is antibiotics; other medications provide supportive care. Without any solid proof, chest PT has been utilized extensively as an adjuvant treatment for adult pneumonia. Chest physiotherapy was found to have a positive effect on fewer days spent in the hospital among older patients who had no history of chronic respiratory disease [10]. Another study revealed that elderly patients were unable to control the release of secretions using passive methods like vibration and postural drainage, which is also referred to as conventional physical therapy (CPT) [11-13]. On the other hand, a different study demonstrated that CPT could raise oxygenation and decrease secretions [14,15]. Postural drainage breathing exercises and mobility exercises were introduced to the chest therapy program after three days of admission. From the perspective of physiotherapy, patients with pneumonia experience a range of disorders, including difficulty clearing mucus, altered breathing patterns, and altered posture, which leads to disruption of daily activities. In a study using pulmonary rehabilitation, which consists of aerobic training, streamlined strength training, and breathing exercises, researchers demonstrated that people with bronchopneumonia have negative short- and long-term limitations in their physical performance. The mMRC Dyspnea Scale, Numerical Pain Rating Scale (NPRS), and Intensive Care Unit (ICU) Mobility Scale were used as outcome measures to evaluate the patient's treatment [16]. Sessions of physiotherapy were split into two halves. To stress the importance of following the recommended action plan, informed consent was requested for the treatment technique when initially the patient was informed about his state. To lessen airway resistance and enhance breathing, we concentrated on clearing obstructive tracheobronchial secretions.

For a week, nebulization with Budecort was administered three times a day to facilitate mucus discharge and enhance the lung's airway. There was a noticeable improvement on every metric during these days, according to the variance in scores. Early mobility of hospitalized patients with pneumonia reduces the length of stay and leverages institutional resources without increasing the risk of outcome measurements [17]. Peripheral oxygen saturation levels improved more when chest PT (continuous positive airway pressure (CPAP)) and traditional chest physiotherapy were used [18]. The majority of hospitals and community centers will implement pulmonary rehabilitation. Patient knowledge of their benefits will encourage more participation and enhance functional traits and quality of life [19,20].

## Conclusions

Patients who require artificial respiration due to severe bronchopneumonia are more prone to experience exercise intolerance and respiratory muscle weakness. Improving their physical function requires early intervention, starting during the acute illness phase. An early start of chest physiotherapy is essential for the recovery phase for treating bronchopneumonia by improving hypersecretion and lung diseases' airway clearance. The goal was to improve the patient's respiratory state and hasten their recovery. Improved gas exchange and less breathing effort are two benefits of increased airway clearance. However, as a result of our meticulously planned pulmonary rehabilitation, the patient's cough severity, dyspnea, and weakness all significantly improved. It is beneficial in increasing functional abilities and enhancing quality of life.

## References

[1] (2017). Estimates of the global, regional, and national morbidity, mortality, and aetiologies of lower respiratory tract infections in 195 countries: a systematic analysis for the Global Burden of Disease Study 2015. Lancet Infect Dis.

[2] White RJ, Blainey AD, Harrison KJ, Clarke SK (1981). Causes of pneumonia presenting to a district general hospital. Thorax.

[3] Jones RN (2010). Microbial etiologies of hospital-acquired bacterial pneumonia and ventilator-associated bacterial pneumonia. Clin Infect Dis.

[4] Fernández-Sabé N, Carratalà J, Rosón B, Dorca J, Verdaguer R, Manresa F, Gudiol F (2003). Community-acquired pneumonia in very elderly patients: causative organisms, clinical characteristics, and outcomes. Medicine (Baltimore).

[5] Prendki V, Scheffler M, Huttner B (2018). Low-dose computed tomography for the diagnosis of pneumonia in elderly patients: a prospective, interventional cohort study. Eur Respir J.

[6] SK D, A D, S K, De P, S C, Samanta A, Mukherjee A (2009). Ethnobotanical study in a remote district of West Bengal, India. Pharmbit.

[7] Torres A, Blasi F, Peetermans WE, Viegi G, Welte T (2014). The aetiology and antibiotic management of community-acquired pneumonia in adults in Europe: a literature review. Eur J Clin Microbiol Infect Dis.

[8] Chigira Y, Takai T, Igusa H, Dobashi K (2015). Effects of early physiotherapy with respect to severity of pneumonia of elderly patients admitted to an intensive care unit: a single center study in Japan. J Phys Ther Sci.

[9] Clini E, Ambrosino N (2005). Early physiotherapy in the respiratory intensive care unit. Respir Med.

[10] Pruitt B, Jacobs M (2005). Clearing away pulmonary secretions. Nursing.

[11] Pusey-Reid E (2014). Preventing postoperative pneumonia. Nurs Crit Care.

[12] Zisi D, Chryssanthopoulos C, Nanas S, Philippou A (2022). The effectiveness of the active cycle of breathing technique in patients with chronic respiratory diseases: a systematic review. Heart Lung.

[13] Tseng CN, Chen CC, Wu SC, Lin LC (2007). Effects of a range-of-motion exercise programme. J Adv Nurs.

[14] Yadav V, Naqvi WM, Burhani TS (2020). Pandemics and physiotherapy: an overview of the role of the physiotherapists in restoring functions and quality of life. Int J Res Pharm Sci.

[15] Lestari NE, Nurhaeni N, Chodidjah S (2018). The combination of nebulization and chest physiotherapy improved respiratory status in children with pneumonia. Enferm Clínica.

[16] Nandanwar RR, Singh R, Karanjkar SM, Bhagwani RS (2022). The impact of pulmonary rehabilitation in a case of acute respiratory distress syndrome with bronchopneumonia: a case report. Cureus.

[17] Mundy LM, Leet TL, Darst K, Schnitzler MA, Dunagan WC (2003). Early mobilization of patients hospitalized with community-acquired pneumonia. Chest.

[18] Chaves GS, Freitas DA, Santino TA, Nogueira PA, Fregonezi GA, Mendonça KM (2019). Chest physiotherapy for pneumonia in children. Cochrane Database Syst Rev.

[19] Schelling G, Stoll C, Vogelmeier C (2000). Pulmonary function and health-related quality of life in a sample of long-term survivors of the acute respiratory distress syndrome. Intensive Care Med.

[20] Hsieh MJ, Lee WC, Cho HY (2018). Recovery of pulmonary functions, exercise capacity, and quality of life after pulmonary rehabilitation in survivors of ARDS due to severe influenza A (H1N1) pneumonitis. Influenza Other Respir Viruses.

